# Early relative glycemic stress trajectories identify a high-risk metabolic phenotype in critically ill patients with atherosclerotic cardiovascular disease: a dual-cohort study

**DOI:** 10.3389/fendo.2026.1909554

**Published:** 2026-08-14

**Authors:** You Wu, Kangxing Wang

**Affiliations:** Department of Critical Care Medicine, West China Hospital, Sichuan University, Chengdu, Sichuan, China

**Keywords:** atherosclerotic cardiovascular disease, critical illness, glycemic trajectories, machine learning, stress hyperglycemia ratio

## Abstract

**Background:**

The stress hyperglycemia ratio (SHR) captures acute glucose elevation relative to chronic glycemic background. However, the prognostic value of early dynamic SHR patterns in critically ill patients with atherosclerotic cardiovascular disease (ASCVD) remains uncertain.

**Methods:**

We conducted a retrospective dual-cohort study using MIMIC-IV v3.1 for trajectory development and NWICU v0.1.0 for external validation. Adults with ASCVD, available HbA1c measurements, and valid SHR values in at least three of six 12-hour windows during the first 72 hours after ICU admission were included. Trajectories were derived in MIMIC-IV using group-based trajectory modeling, and NWICU patients were assigned to the closest MIMIC-IV-derived trajectory. The primary outcome was 28-day all-cause mortality. Cox models adjusted for demographic characteristics, ICU type, comorbidities, severity and functional scores, vital signs, laboratory measurements, and early interventions. Sensitivity, subgroup, and exploratory machine-learning analyses were also performed.

**Results:**

The study included 8,835 patients from MIMIC-IV and 1,705 from NWICU. Three early SHR trajectories were identified: moderate-stable, low-declining, and persistently high. The persistently high trajectory was the largest class in both cohorts and showed the poorest 28-day survival. In MIMIC-IV, the fully adjusted hazard ratio for Class 3 versus Class 1 was 1.37 (95% CI 1.15-1.64). The association was also observed in NWICU (HR 2.04, 95% CI 1.39-3.01). Findings for ICU and in-hospital mortality were directionally concordant. HbA1c and first-12-hour maximum glucose were the leading predictors of persistently high trajectory membership.

**Conclusions:**

Early SHR trajectories identified a common high-risk metabolic phenotype in critically ill patients with ASCVD. The persistently high trajectory reflected acute glycemic deviation from the HbA1c-defined background and was independently associated with short-term mortality. Dynamic SHR profiling may support metabolic risk stratification in cardiovascular critical illness.

## Background

Atherosclerotic cardiovascular disease (ASCVD), principally coronary heart disease and ischemic stroke, remains a leading contributor to global mortality and disability (1). In critically ill patients, established ASCVD represents a vulnerable cardiovascular substrate exposed to acute inflammation, hemodynamic stress, neuroendocrine activation, renal dysfunction, and organ-support interventions (2). In this setting, metabolic stress may be more than a laboratory abnormality; it may reflect acute systemic decompensation superimposed on chronic vascular disease.

Disordered glucose metabolism is common during critical illness. Stress hyperglycemia is driven by catecholamine and cortisol release, inflammatory signaling, increased hepatic glucose production, and peripheral insulin resistance (3). However, absolute glucose values are difficult to interpret without chronic glycemic context. The same glucose concentration may indicate marked acute metabolic stress in a patient with near-normal baseline glycemia, but a smaller relative perturbation in a patient with long-standing hyperglycemia (4). This distinction is particularly relevant in ASCVD, where chronic diabetes and acute stress hyperglycemia may represent biologically and prognostically different states. The stress hyperglycemia ratio (SHR), calculated as acute glucose divided by HbA1c-derived estimated average glucose, was proposed to quantify relative hyperglycemia after accounting for preadmission glycemic exposure (5, 6). HbA1c itself is associated with cardiovascular risk, but SHR addresses a different question: how far the current glucose state deviates from the patient’s chronic glycemic background (7). Prior studies have shown that admission glucose, persistent hyperglycemia, and glycemic variability are associated with adverse outcomes after acute myocardial infarction and in critical illness (8, 9). Nevertheless, most SHR studies have evaluated a single baseline value, leaving the temporal pattern of relative glycemic stress insufficiently characterized. Group-based trajectory modeling can identify latent patient subgroups that share similar longitudinal biomarker patterns (10). In critical-care research, trajectory-based phenotyping has revealed clinically meaningful heterogeneity in vital signs and temperature patterns that is not apparent from baseline measurements alone (11, 12). We therefore hypothesized that early SHR trajectories during the first 72 hours after ICU admission could define dynamic relative glycemic stress phenotypes in critically ill patients with ASCVD. We further tested whether a high SHR trajectory would identify a risk phenotype distinct from chronic diabetes or absolute hyperglycemia, whether this phenotype would be supported in an external cohort, and whether it could be recognized early using routinely available clinical variables.

## Methods

### Data sources

This retrospective cohort study used two publicly available critical care databases. The development cohort was derived from MIMIC-IV version 3.1, which contains de-identified electronic health records for adult ICU admissions at Beth Israel Deaconess Medical Center between 2008 and 2022 (13). External validation was performed in NWICU version 0.1.0, a harmonized multi-hospital ICU database from Chicago covering admissions between 2020 and 2022 (14). An authorized researcher (Kangxing Wang; PhysioNet Credential ID: 13024213) obtained access after completing the required human-subjects research training and data-use agreements.

### Study population

**Figure 1:**
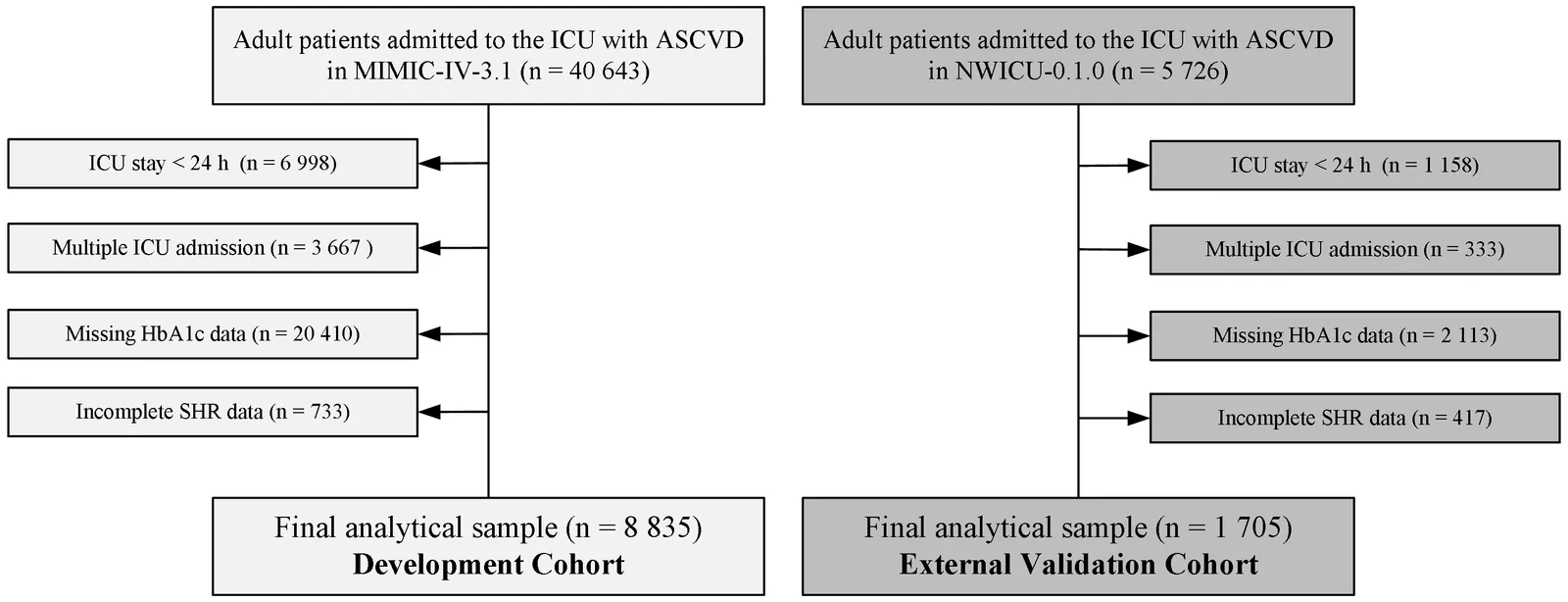
Patient selection flowchart for the development and external validation cohorts. ASCVD, atherosclerotic cardiovascular disease; HbA1c, glycated hemoglobin; ICU, intensive care unit; MIMIC-IV, Medical Information Mart for Intensive Care IV; NWICU, Northwestern ICU database; SHR, stress hyperglycemia ratio.

Adult ICU patients with ASCVD were eligible. ASCVD was identified using structured ICD-9 and ICD-10 discharge diagnosis codes and was defined as coronary heart disease, acute myocardial infarction, ischemic stroke, or related atherosclerotic cardiovascular conditions. For patients with multiple ICU admissions, only the first ICU admission was retained. Patients were excluded if ICU length of stay was less than 24 hours, HbA1c data were missing, or valid SHR values were available in fewer than three of the six 12-hour windows during the first 72 hours after ICU admission. After applying these criteria, 8835 patients from MIMIC-IV and 1705 patients from NWICU were included (**Figure 1**).

### Exposure definition and trajectory modeling

SHR was calculated as blood glucose (mg/dL) divided by HbA1c-derived estimated average glucose: SHR = blood glucose/[28.7 × HbA1c (%) - 46.7] (3). During the first 72 hours after ICU admission, glucose values were grouped into six 12-hour windows, and the maximum glucose value in each window was used to calculate window-specific SHR. To quantify glucose-monitoring intensity and address potential bias arising from differential measurement frequency, glucose-monitoring frequency was defined as the number of distinct glucose sampling time points recorded during the first 72 hours after ICU admission. Multiple glucose records sharing the same sampling time were counted as a single measurement. Group-based trajectory modeling was performed in MIMIC-IV to identify latent SHR patterns (10). Candidate models with one to seven classes were compared using information criteria, posterior classification probabilities, odds of correct classification, class proportions, and clinical interpretability. In NWICU, each patient was assigned to the closest MIMIC-IV-derived trajectory by minimizing the mean squared error between the observed SHR profile and class-specific fitted trajectories (15).

### Covariates

Baseline covariates were extracted primarily within the first 24 hours after ICU admission. They included demographics, ICU type, severity scores, comorbidities, vital signs, laboratory tests, blood gas parameters, glucose-related variables, glucose-monitoring frequency, and early organ-support interventions. Specific variables included age, sex, race, SOFA, OASIS, Glasgow Coma Scale, Charlson Comorbidity Index, hypertension, diabetes, chronic obstructive pulmonary disease, heart failure, stroke, liver disease, chronic kidney disease, malignancy, ischemic stroke, coronary heart disease, acute myocardial infarction, atrial fibrillation, mean arterial pressure, heart rate, respiratory rate, temperature, hemoglobin, white blood cell count, platelet count, alanine aminotransferase, aspartate aminotransferase, total bilirubin, albumin, creatinine, international normalized ratio, activated partial thromboplastin time, pH, lactate, partial pressure of oxygen, partial pressure of carbon dioxide, bicarbonate, sodium, potassium, calcium, chloride, maximum first-12-hour glucose, HbA1c, baseline SHR, the number of distinct glucose sampling time points during the first 72 hours, mechanical ventilation, renal replacement therapy, vasopressor use, and insulin therapy.

### Outcomes

The primary outcome was 28-day all-cause mortality after ICU admission. Secondary outcomes were ICU mortality and in-hospital mortality. Survival time was censored at 28 days for time-to-event analyses. For the day-3 landmark analyses, the study population was restricted to patients who were alive and under follow-up at 72 hours after ICU admission. The time origin was reset to the 72-hour landmark, and patients were followed from day 3 until death or administrative censoring at day 28.

### Statistical analysis

Continuous variables were summarized as medians with interquartile ranges, and categorical variables were summarized as counts with percentages. Baseline characteristics were compared across trajectory classes using nonparametric tests for continuous variables and chi-square or Fisher exact tests for categorical variables, as appropriate. Standardized mean differences were used to quantify between-class imbalance.

HbA1c was required to calculate SHR and was therefore not imputed. Patients without HbA1c were excluded from the analytic cohort. To assess potential selection related to HbA1c availability, baseline characteristics and outcomes were compared between eligible ASCVD ICU patients with and without HbA1c measurements in MIMIC-IV before HbA1c-based exclusion. For other covariates, variables with more than 30% missingness were not included in multivariable analyses, whereas variables with 30% or less missingness were imputed using multiple imputation by chained equations, generating 10 imputed datasets for each cohort. Regression estimates from imputed datasets were pooled according to Rubin’s rules.

Kaplan–Meier curves and log-rank tests were used to compare unadjusted 28-day survival across SHR trajectory classes. Cox proportional hazards models estimated hazard ratios (HRs) and 95% confidence intervals (CIs) for 28-day mortality, with Class 1 as the reference. Model 1 was unadjusted. Model 2 adjusted for age, sex, and race. Model 3 further adjusted for age, sex, race, ICU type, comorbidities, severity and functional scores, vital signs, laboratory measurements, and early interventions. Day-3 landmark Kaplan–Meier and Cox analyses were performed among patients who remained alive at 72 hours, with survival time recalculated from the landmark to day 28 using the same sequential adjustment strategy. For the day-3 landmark analysis, Model 4 additionally adjusted for log-transformed glucose-monitoring frequency during the first 72 hours. Logistic regression models using the same three-model sequential adjustment strategy evaluated ICU mortality and in-hospital mortality. Prespecified subgroup analyses assessed effect modification on the multiplicative scale by including interaction terms between SHR trajectory class and subgroup variables in the Cox models.

As an exploratory analysis, supervised machine-learning models were developed to predict Class 3 membership using variables available within the first 24 hours. The MIMIC-IV cohort was divided by stratified random sampling into a training set and an internal validation set at a 7:3 ratio. Feature selection and model development were performed only in the training set to avoid information leakage. Feature selection used Boruta and LASSO, and model reporting followed general prediction-model reporting principles (16, 17). XGBoost, gradient boosting machine, random forest, support vector machine, and K-nearest neighbors were trained in the MIMIC-IV training set (18–22). Hyperparameters were optimized to maximize the cross-validated AUC using repeated 10-fold cross-validation with three repeats within the MIMIC-IV training set. Model performance was evaluated in both the MIMIC-IV internal validation set and the NWICU external validation cohort using AUC, sensitivity, specificity, precision, F1-score, and Matthews correlation coefficient. SHAP was used to interpret the XGBoost model. To facilitate reproducible implementation of the final XGBoost model, an interactive web-based calculator was developed using R Shiny. Based on the same 33 predictors, the calculator returns the estimated probability of Class 3 SHR trajectory membership and the corresponding model-assigned class using the prespecified threshold of 0.50. The calculator is intended for research use and is publicly accessible at https://wangkangxing.shinyapps.io/shr-class3-calculator/. All analyses were performed using R software, version 4.4.2 (R Foundation for Statistical Computing, Vienna, Austria). A two-sided P value < 0.05 was considered statistically significant.

## Results

### Patient selection and trajectory identification

**Figure 2:**
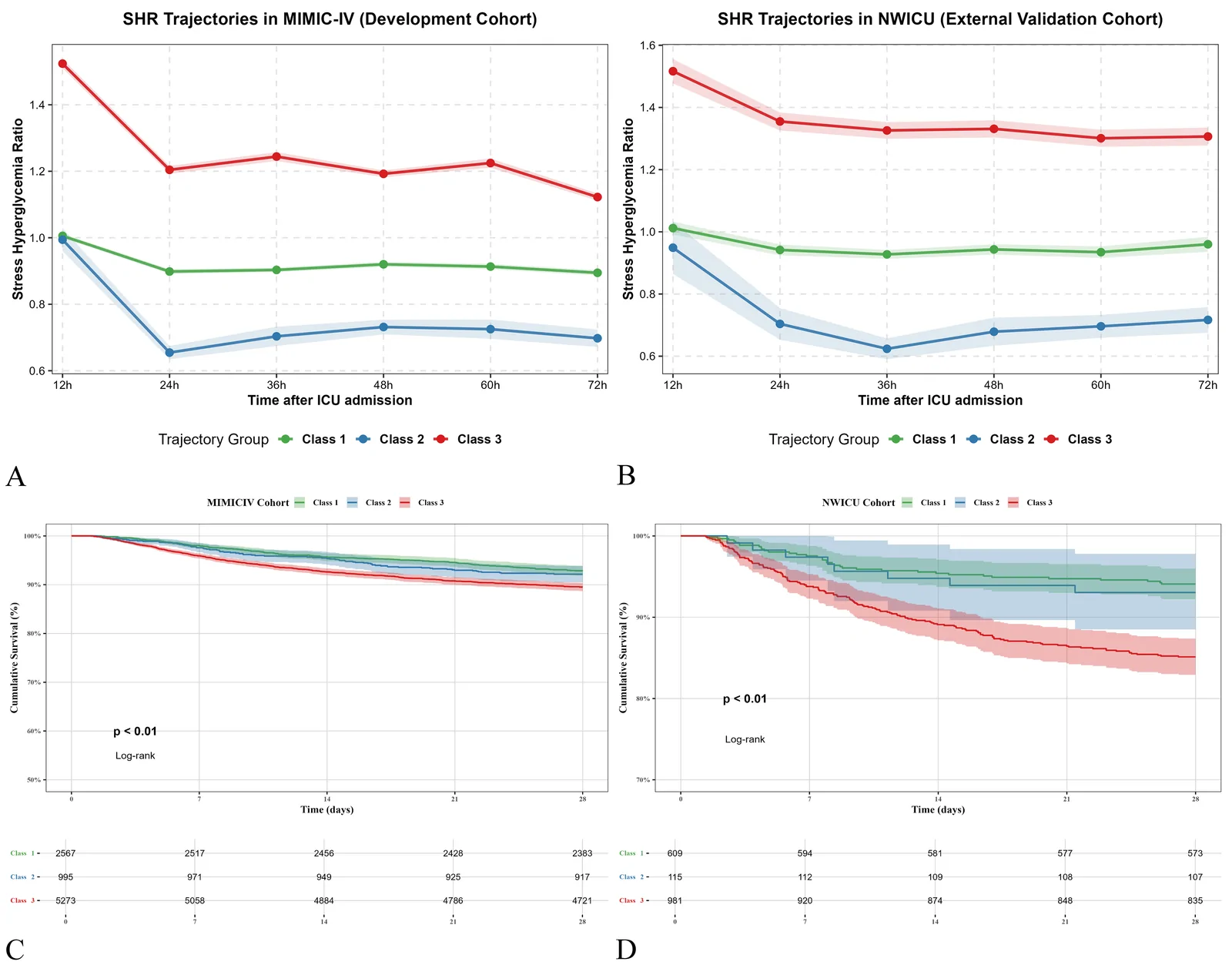
Early SHR trajectories and 28-day survival in the development and external validation cohorts. **(A)** Early SHR trajectories in the MIMIC-IV development cohort. **(B)** Corresponding trajectories in the NWICU external validation cohort after minimum mean squared error assignment. Class 1 represents the moderate-stable SHR trajectory, Class 2 the low-declining SHR trajectory, and Class 3 the persistently high SHR trajectory. **(C, D)** Kaplan-Meier curves for 28-day cumulative survival in MIMIC-IV and NWICU, respectively. Differences were assessed using log-rank tests. ICU, intensive care unit; MIMIC-IV, Medical Information Mart for Intensive Care IV; NWICU, Northwestern ICU database; SHR, stress hyperglycemia ratio.

In the MIMIC-IV development cohort, group-based trajectory modeling identified three distinct early SHR trajectories during the first 72 hours after ICU admission (**Figure 2A**). The three-class model was selected after jointly considering model fit, classification quality, class size, and clinical interpretability. The selected model showed acceptable Bayesian information criterion performance among candidate solutions, adequate class size (>10% of the cohort in each class), and average posterior probability >0.70 for all classes (**Supplementary Table 1**). Although models with more trajectory classes were explored, they yielded smaller or less clinically stable classes; therefore, the three-class solution was retained for external assignment and outcome analyses. Before HbA1c-based exclusion, 9,568 of 29,978 eligible ASCVD ICU patients in MIMIC-IV had HbA1c measurements. Baseline comparisons indicated measurable differences between patients with and without HbA1c measurements, supporting cautious interpretation of generalizability (**Supplementary Table 2**).

Class 1 included 2,567 patients (29.1%) and followed the moderate-stable SHR trajectory, with SHR values remaining close to 0.9-1.0 after the first 24 hours. Class 2 included 995 patients (11.3%) and followed the low-declining SHR trajectory. Class 3 included 5,273 patients (59.7%) and followed the persistently high SHR trajectory, beginning at approximately 1.5 and remaining above the other classes throughout follow-up. When the MIMIC-IV-derived trajectory patterns were applied to the NWICU cohort using minimum mean squared error assignment, similar early SHR patterns were observed (**Figure 2B**), supporting the descriptive transportability of the trajectory structure.

### Baseline characteristics

Baseline characteristics across SHR trajectory classes are summarized in **Table 1**. The trajectory groups differed in both chronic glycemic background and acute glycemic status. Patients in Class 3 had the highest baseline SHR (1.42 [1.23, 1.68]) and higher first-12-hour maximum glucose than Class 1, whereas patients in Class 2 had the highest prevalence of diabetes (76.3%) and the highest HbA1c level (8.70% [7.30, 10.50]). Glucose-monitoring frequency also differed across trajectory classes, with median 72-hour measurement counts of 5 (3, 8), 8 (5, 11), and 9 (6, 12) in Classes 1, 2, and 3, respectively (P < 0.001). Compared with Class 1, Class 3 had higher illness severity and greater use of early organ-support interventions, including mechanical ventilation and vasopressors. Observed 28-day, ICU, and in-hospital mortality were also higher in Class 3 than in the other trajectory groups.

**Table 1:** Baseline characteristics of the MIMIC-IV development cohort according to SHR trajectory class.

Variable	Overall	Class1	Class2	Class3	*P* value	SMD
	(N=8835)	(N=2567)	(N=995)	(N=5273)		
Demographics						
Age, y	70.00 [61.00, 78.00]	70.00 [60.00, 79.00]	67.00 [58.00, 76.00]	70.00 [62.00, 78.00]	**<0.001**	**0.132**
Gender (Male), n (%)	5755 (65.1)	1608 (62.6)	604 (60.7)	3543 (67.2)	**<0.001**	0.090
Race, n (%)					**<0.001**	**0.194**
Asian	196 (2.2)	70 (2.7)	24 (2.4)	102 (1.9)		
Black	699 (7.9)	229 (8.9)	143 (14.4)	327 (6.2)		
Other	2453 (27.8)	680 (26.5)	276 (27.7)	1497 (28.4)		
White	5487 (62.1)	1588 (61.9)	552 (55.5)	3347 (63.5)		
**ICU Type, n (%)**					**<0.001**	**0.412**
CCU	5462 (61.8)	1233 (48.0)	614 (61.7)	3615 (68.6)		
Medical ICU	1026 (11.6)	244 (9.5)	183 (18.4)	599 (11.4)		
Neuro ICU	1103 (12.5)	548 (21.3)	85 (8.5)	470 (8.9)		
Other ICU	175 (2.0)	87 (3.4)	17 (1.7)	71 (1.3)		
Surgical ICU	1069 (12.1)	455 (17.7)	96 (9.6)	518 (9.8)		
Severity of illness						
SOFA	4.00 [2.00, 7.00]	3.00 [2.00, 5.00]	5.00 [3.00, 7.00]	5.00 [3.00, 7.00]	**<0.001**	**0.357**
OASIS	31.00 [26.00, 37.00]	30.00 [25.00, 35.00]	32.00 [27.00, 38.00]	32.00 [27.00, 38.00]	**<0.001**	**0.211**
GCS	15.00 [14.00, 15.00]	15.00 [13.00, 15.00]	15.00 [13.00, 15.00]	15.00 [14.00, 15.00]	**<0.001**	0.054
Charlson	6.00 [4.00, 8.00]	6.00 [4.00, 7.00]	6.00 [4.00, 8.00]	5.00 [4.00, 8.00]	**<0.001**	**0.168**
Comorbidities						
Hypertension, n (%)	4191 (47.4)	1306 (50.9)	430 (43.2)	2455 (46.6)	**<0.001**	**0.103**
Diabetes, n (%)	3564 (40.3)	928 (36.2)	759 (76.3)	1877 (35.6)	**<0.001**	**0.598**
COPD, n (%)	667 (7.5)	175 (6.8)	72 (7.2)	420 (8.0)	0.181	0.029
HF, n (%)	3326 (37.6)	834 (32.5)	418 (42.0)	2074 (39.3)	**<0.001**	**0.132**
Stroke, n (%)	2057 (23.3)	921 (35.9)	154 (15.5)	982 (18.6)	**<0.001**	**0.320**
Liver Disease, n (%)	182 (2.1)	38 (1.5)	20 (2.0)	124 (2.4)	**0.039**	0.042
CKD, n (%)	2109 (23.9)	528 (20.6)	326 (32.8)	1255 (23.8)	**<0.001**	**0.185**
Malignancy, n (%)	882 (10.0)	239 (9.3)	94 (9.4)	549 (10.4)	0.261	0.025
Ischemic Stroke, n (%)	1823 (20.6)	828 (32.3)	146 (14.7)	849 (16.1)	**<0.001**	**0.283**
CHD, n (%)	3719 (42.1)	806 (31.4)	439 (44.1)	2474 (46.9)	**<0.001**	**0.214**
AMI, n (%)	2218 (25.1)	505 (19.7)	259 (26.0)	1454 (27.6)	**<0.001**	**0.125**
AF, n (%)	3589 (40.6)	936 (36.5)	334 (33.6)	2319 (44.0)	**<0.001**	**0.143**
Vital signs						
MAP, mmHg	60.00 [54.00, 67.00]	62.00 [56.00, 71.00]	59.00 [53.00, 65.00]	59.00 [53.00, 65.00]	**<0.001**	**0.231**
Heart rate, beats/min	97.00 [87.00, 109.00]	95.00 [85.00, 107.00]	98.00 [88.00, 109.00]	97.00 [87.00, 109.00]	**<0.001**	0.085
Respiratory rate, times/min	27.00 [24.00, 31.00]	26.00 [24.00, 30.00]	27.00 [24.00, 31.00]	27.00 [24.00, 31.00]	**<0.001**	0.089
Temperature, °C	37.17 [36.94, 37.60]	37.17 [36.94, 37.56]	37.17 [36.94, 37.60]	37.20 [36.94, 37.61]	0.092	0.041
Laboratory						
Hb, g/dL	10.80 [9.00, 12.60]	11.50 [9.80, 13.10]	10.20 [8.60, 12.00]	10.50 [8.80, 12.40]	**<0.001**	**0.301**
Wbc, 10^9/L	11.10 [8.40, 14.80]	10.10 [7.70, 13.00]	10.90 [8.10, 14.70]	11.80 [8.80, 15.60]	**<0.001**	**0.236**
Plt, 10^9/L	177.00 [132.00, 237.00]	196.00 [147.50, 252.00]	182.00 [136.00, 245.00]	168.00 [126.00, 226.00]	**<0.001**	**0.175**
ALT, U/L	22.00 [14.00, 40.00]	21.00 [14.00, 36.00]	21.00 [14.00, 39.00]	22.00 [14.00, 43.00]	**0.001**	0.063
AST, U/L	34.00 [22.00, 65.00]	30.00 [20.00, 55.00]	32.00 [21.00, 58.00]	37.00 [22.00, 72.00]	**<0.001**	**0.076**
Tbi, mg/dL	0.60 [0.40, 0.90]	0.60 [0.40, 0.80]	0.50 [0.30, 0.70]	0.60 [0.40, 0.90]	**<0.001**	**0.141**
Alb, g/dL	3.30 [2.90, 3.70]	3.50 [3.10, 3.90]	3.20 [2.70, 3.50]	3.30 [2.90, 3.70]	**<0.001**	**0.392**
Cre, mg/dL	1.00 [0.80, 1.30]	0.90 [0.70, 1.20]	1.10 [0.80, 1.60]	1.00 [0.80, 1.40]	**<0.001**	**0.174**
INR	1.30 [1.10, 1.50]	1.20 [1.10, 1.40]	1.30 [1.10, 1.50]	1.30 [1.20, 1.60]	**<0.001**	**0.141**
APTT, seconds	30.80 [27.20, 37.70]	30.50 [27.20, 37.10]	30.50 [26.90, 36.30]	30.90 [27.20, 38.40]	**0.016**	0.077
Blood Gas						
PH	7.40 [7.35, 7.44]	7.41 [7.36, 7.44]	7.39 [7.35, 7.44]	7.39 [7.35, 7.44]	**<0.001**	**0.147**
Lactate,mmol/L	1.70 [1.20, 2.40]	1.40 [1.10, 2.00]	1.80 [1.30, 2.50]	1.80 [1.30, 2.60]	**<0.001**	**0.273**
PO2,mmHg	205.00 [98.00, 334.00]	157.00 [90.00, 311.00]	209.00 [98.00, 332.00]	235.00 [104.00, 343.00]	**<0.001**	**0.173**
PCO2,mmHg	40.00 [35.00, 44.00]	39.00 [35.00, 44.00]	40.00 [35.00, 44.00]	40.00 [36.00, 45.00]	**<0.001**	0.080
HCO3, mmol/L	23.00 [21.00, 25.00]	23.00 [22.00, 25.00]	23.00 [21.00, 25.00]	22.00 [20.00, 24.00]	**<0.001**	**0.191**
Na, mmol/L	137.00 [134.00, 140.00]	138.00 [135.00, 140.00]	136.00 [134.00, 139.00]	136.00 [134.00, 139.00]	**<0.001**	**0.240**
K, mmol/L	4.30 [3.90, 4.80]	4.10 [3.80, 4.60]	4.30 [3.90, 4.80]	4.30 [3.90, 4.90]	**<0.001**	**0.171**
Ca, mg/dL	8.40 [8.00, 8.90]	8.60 [8.20, 9.00]	8.40 [8.00, 8.90]	8.40 [8.00, 8.80]	**<0.001**	**0.196**
Cl, mmol/L	105.00 [101.00, 107.00]	104.00 [101.00, 107.00]	104.00 [100.00, 107.00]	105.00 [101.00, 108.00]	**0.003**	0.070
Blood Glucose						
Baseline max BG 12 h, mg/dL	162.00 [128.00, 201.00]	128.00 [106.00, 158.00]	184.00 [135.00, 233.00]	174.00 [146.00, 211.00]	**<0.001**	**0.585**
Hba1c, %	5.90 [5.50, 6.90]	6.00 [5.60, 6.80]	8.70 [7.30, 10.50]	5.80 [5.40, 6.40]	**<0.001**	**1.117**
Baseline SHR	1.23 [0.99, 1.50]	0.99 [0.88, 1.11]	0.87 [0.69, 1.15]	1.42 [1.23, 1.68]	**<0.001**	**0.828**
72-h glucose measurement count, n	8.00 [4.00, 11.00]	5.00 [3.00, 8.00]	8.00 [5.00, 11.00]	9.00 [6.00, 12.00]	**<0.001**	**0.436**
Interventions						
Mechanical Ventilation, n (%)	4410 (49.9)	930 (36.2)	494 (49.6)	2986 (56.6)	**<0.001**	**0.277**
RRT, n (%)	213 (2.4)	33 (1.3)	32 (3.2)	148 (2.8)	**<0.001**	0.087
Vasopressors, n (%)	3908 (44.2)	798 (31.1)	472 (47.4)	2638 (50.0)	**<0.001**	**0.262**
Insulin, n (%)	4718 (53.4)	902 (35.1)	693 (69.6)	3123 (59.2)	**<0.001**	**0.484**
Outcome						
28-day Survival Time (days)	28.00 [28.00, 28.00]	28.00 [28.00, 28.00]	28.00 [28.00, 28.00]	28.00 [28.00, 28.00]	**<0.001**	0.097
Day28outcome, n (%)	814 (9.2)	184 (7.2)	78 (7.8)	552 (10.5)	**<0.001**	0.078
ICU Survival Time (days)	2.72 [1.56, 4.94]	2.53 [1.63, 4.54]	2.48 [1.50, 4.18]	2.87 [1.55, 5.24]	**<0.001**	**0.109**
ICU outcome, n (%)	406 (4.6)	59 (2.3)	35 (3.5)	312 (5.9)	**<0.001**	**0.123**
Hos Survival Time (days)	7.06 [4.96, 12.63]	6.70 [4.41, 11.94]	7.16 [5.14, 11.93]	7.16 [5.09, 13.12]	**<0.001**	0.092
Hos outcome, n (%)	663 (7.5)	120 (4.7)	57 (5.7)	486 (9.2)	**<0.001**	**0.120**

Continuous variables are presented as medians with interquartile ranges, and categorical variables as counts with percentages. P values were calculated using Kruskal-Wallis, chi-square, or Fisher’s exact tests, as appropriate. The 72-hour glucose measurement count represents the number of distinct sampling time points during the first 72 hours.

AF, atrial fibrillation; Alb, albumin; ALT, alanine aminotransferase; AMI, acute myocardial infarction; APTT, activated partial thromboplastin time; ASCVD, atherosclerotic cardiovascular disease; AST, aspartate aminotransferase; BG, blood glucose; Ca, calcium; CHD, coronary heart disease; CKD, chronic kidney disease; Cl, chloride; COPD, chronic obstructive pulmonary disease; Cre, creatinine; GCS, Glasgow Coma Scale; Hb, hemoglobin; HbA1c, glycated hemoglobin; HCO₃, bicarbonate; HF, heart failure; ICU, intensive care unit; INR, international normalized ratio; IQR, interquartile range; K, potassium; MAP, mean arterial pressure; MIMIC-IV, Medical Information Mart for Intensive Care IV; Na, sodium; OASIS, Oxford Acute Severity of Illness Score; PCO₂, partial pressure of carbon dioxide; Plt, platelet count; PO₂, partial pressure of oxygen; RRT, renal replacement therapy; SHR, stress hyperglycemia ratio; SMD, standardized mean difference; SOFA, Sequential Organ Failure Assessment; Tbi, total bilirubin; WBC, white blood cell count. Bold values indicate P < 0.05; bold SMD values indicate SMD ≥ 0.10.

### Association between SHR trajectories and mortality

Kaplan–Meier curves showed separation of 28-day survival across trajectories in both cohorts (log-rank P < 0.01; **Figures 2C, D**). Class 3 consistently had the poorest 28-day survival. In MIMIC-IV, Class 3 was associated with higher 28-day mortality compared with Class 1 in the unadjusted model (HR 1.50, 95% CI 1.27–1.77; P < 0.001) and remained associated after full adjustment (HR 1.37, 95% CI 1.15–1.64; P < 0.001). Class 2 was not significantly associated with 28-day mortality after full adjustment (HR 1.28, 95% CI 0.97–1.69; P = 0.082).

In NWICU, the association between Class 3 and 28-day mortality was also observed. Compared with Class 1, Class 3 was associated with increased mortality in the unadjusted model (HR 2.64, 95% CI 1.83–3.81; P < 0.001) and after full adjustment (HR 2.04, 95% CI 1.39–3.01; P < 0.001). Class 2 again showed no statistically significant association after full adjustment (HR 1.32, 95% CI 0.60–2.91; P = 0.485) (**Table 2**).

**Table 2:** Associations between SHR trajectory classes and 28-day all-cause mortality in the development and external validation cohorts.

Variable	Model 1		Model 2		Model 3	
	HR (95% CI)	*P* value	HR (95% CI)	*P* value	HR (95% CI)	*P* value
MIMIC-IV						
Class 2 vs Class 1	1.10 (0.84–1.44)	0.477	1.21 (0.92–1.57)	0.167	1.28 (0.97–1.69)	0.082
Class 3 vs Class 1	1.50 (1.27–1.77)	**<0.001**	1.56 (1.32–1.84)	**<0.001**	1.37 (1.15–1.64)	**<0.001**
NWICU						
Class 2 vs Class 1	1.18 (0.55–2.56)	0.669	1.33 (0.61–2.88)	0.471	1.32 (0.60–2.91)	0.485
Class 3 vs Class 1	2.64 (1.83–3.81)	**<0.001**	2.54 (1.76–3.67)	**<0.001**	2.04 (1.39–3.01)	**<0.001**

Model 1 was unadjusted.

Model 2 adjusted for age, sex, and race.

Model 3 adjusted for age, sex, race, ICU type, comorbidities including stroke, hypertension, diabetes, chronic obstructive pulmonary disease, heart failure, liver disease, chronic kidney disease, malignancy, ischemic stroke, coronary heart disease, acute myocardial infarction, and atrial fibrillation; severity and functional scores including Charlson Comorbidity Index, SOFA score, and Glasgow Coma Scale; vital signs including minimum mean arterial pressure, maximum heart rate, maximum respiratory rate, and maximum temperature within the first 24 hours; laboratory measurements including hemoglobin, white blood cell count, platelet count, creatinine, international normalized ratio, activated partial thromboplastin time, pH, lactate, bicarbonate, sodium, potassium, chloride, and calcium; and early interventions including mechanical ventilation, renal replacement therapy, vasopressor use, and insulin therapy.

APTT, activated partial thromboplastin time; CI, confidence interval; GCS, Glasgow Coma Scale; HR, hazard ratio; ICU, intensive care unit; INR, international normalized ratio; MAP, mean arterial pressure; MIMIC-IV, Medical Information Mart for Intensive Care IV; NWICU, Northwestern ICU database; RRT, renal replacement therapy; SHR, stress hyperglycemia ratio; SOFA, Sequential Organ Failure Assessment. Bold values indicate P < 0.05.

Sensitivity analyses using ICU mortality and in-hospital mortality were directionally concordant for Class 3 (**Supplementary Table 3**). Compared with Class 1, Class 3 was associated with higher ICU mortality in MIMIC-IV after full adjustment (OR 2.07, 95% CI 1.51–2.82; P < 0.001) and in NWICU (OR 2.55, 95% CI 1.34–4.85; P = 0.004). Class 3 was also associated with higher in-hospital mortality in MIMIC-IV (OR 1.83, 95% CI 1.45–2.32; P < 0.001) and NWICU (OR 1.88, 95% CI 1.34–2.64; P < 0.001). Associations for Class 2 varied across secondary outcomes, including higher ICU mortality in MIMIC-IV (OR 1.71, 95% CI 1.08–2.72; P = 0.023) and higher in-hospital mortality in NWICU (OR 2.24, 95% CI 1.24–4.07; P = 0.008), but were not consistent across cohorts or outcomes.

### Day-3 landmark analysis

Among patients included in the day-3 landmark cohort, post-landmark survival differed significantly across SHR trajectory classes in both MIMIC-IV and NWICU (both log-rank P < 0.01; **Supplementary Figure 1**). In MIMIC-IV, Class 3 remained associated with higher 28-day mortality after multivariable adjustment (Model 3: HR 1.36, 95% CI 1.13–1.64; P = 0.001), and the association was materially unchanged after additional adjustment for the log-transformed 72-hour glucose measurement count (Model 4: HR 1.33, 95% CI 1.10–1.61; P = 0.003). Consistent results were observed in NWICU (Model 3: HR 2.14, 95% CI 1.41–3.25; P < 0.001; Model 4: HR 2.21, 95% CI 1.45–3.36; P < 0.001). Class 2 was not significantly associated with post-landmark mortality in either cohort. These findings indicate that the association between the Class 3 trajectory and mortality remained robust after accounting for survival to day 3 and glucose monitoring frequency (**Table 3**).

**Table 3:** Day-3 landmark associations between SHR trajectory classes and 28-day all-cause mortality in the development and external validation cohorts.

Variable	Model 1		Model 2		Model 3		Model 4	
	HR (95% CI)	*P* value	HR (95% CI)	*P* value	HR (95% CI)	*P* value	HR (95% CI)	*P* value
MIMIC-IV								
Class 2 vs Class 1	1.06 (0.80–1.40)	0.678	1.16 (0.88–1.53)	0.299	1.24 (0.93–1.66)	0.139	1.23 (0.92–1.65)	0.157
Class 3 vs Class 1	1.40 (1.18–1.67)	**<0.001**	1.47 (1.23–1.74)	**<0.001**	1.36 (1.13–1.64)	**0.001**	1.33 (1.10–1.61)	**0.003**
NWICU								
Class 2 vs Class 1	1.20 (0.53–2.75)	0.66	1.36 (0.59–3.11)	0.47	1.35 (0.58–3.15)	0.481	1.35 (0.58–3.14)	0.489
Class 3 vs Class 1	2.68 (1.80–3.98)	**<0.001**	2.58 (1.74–3.84)	**<0.001**	2.14 (1.41–3.25)	**<0.001**	2.21 (1.45–3.36)	**<0.001**

The landmark analysis included patients alive at 72 hours, with follow-up calculated from day 3. Models 1–3 followed the adjustment strategy used in **Table 2**. Model 4 additionally adjusted for ln(1 + 72-hour glucose measurement count). Estimates from multiply imputed datasets were pooled using Rubin’s rules.

CI, confidence interval; HR, hazard ratio; ICU, intensive care unit; MIMIC-IV, Medical Information Mart for Intensive Care IV; NWICU, Northwestern ICU database; SHR, stress hyperglycemia ratio. Bold values indicate P < 0.05.

### Subgroup analyses

**Figure 3:**
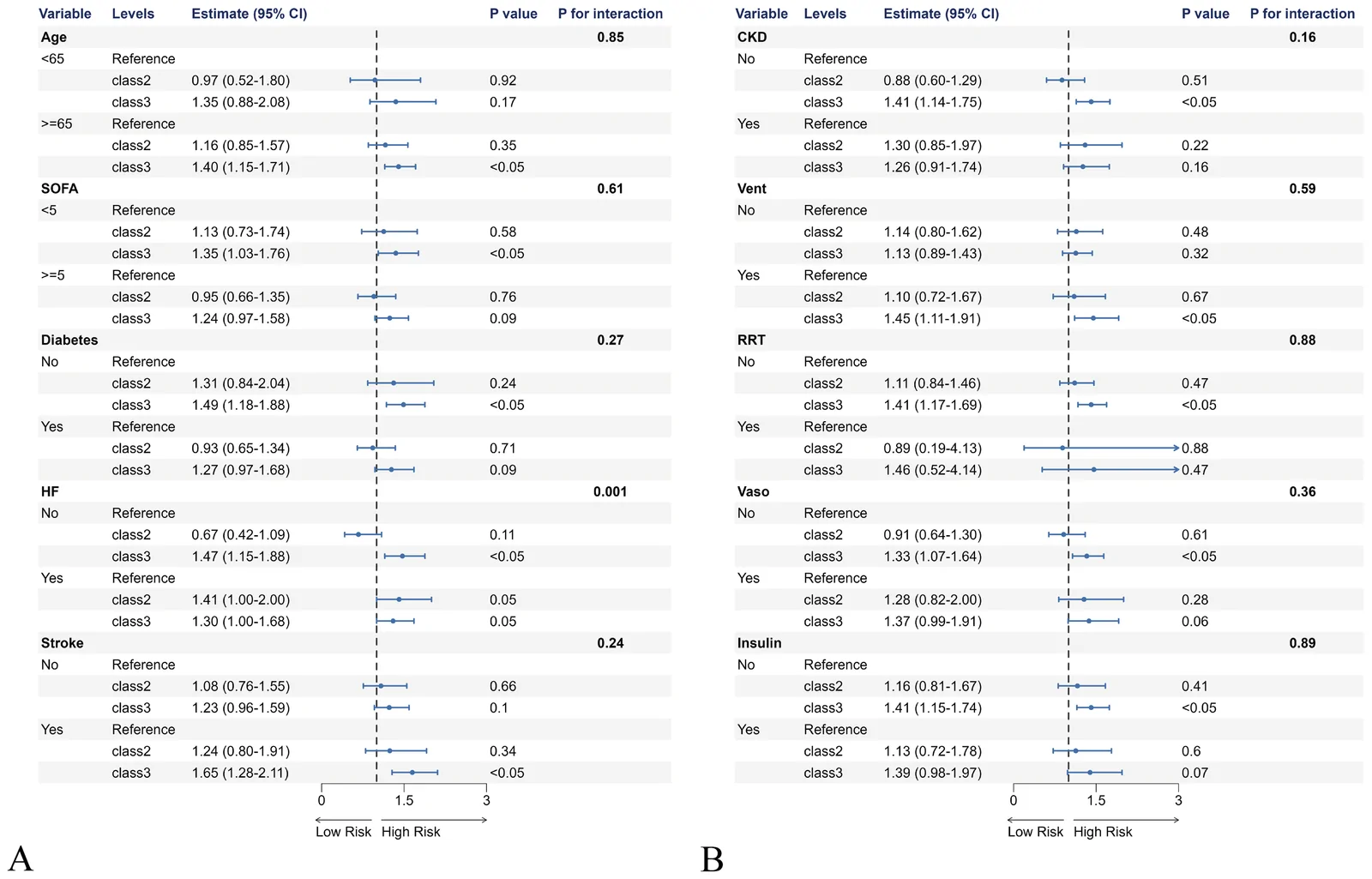
**(A)** Subgroup analyses stratified by age, SOFA score, diabetes, heart failure, and stroke. **(B)** Subgroup analyses stratified by chronic kidney disease, mechanical ventilation, renal replacement therapy, vasopressor use, and insulin use.

Prespecified subgroup analyses showed that the association between Class 3 and 28-day mortality was generally consistent across clinically relevant strata (**Figure 3**). No significant interactions were observed for age, SOFA strata, diabetes, stroke, chronic kidney disease, mechanical ventilation, renal replacement therapy, vasopressor use, or insulin therapy. Heart failure showed a significant interaction (P for interaction = 0.001), suggesting a potential difference in the association across heart failure status.

### Machine-learning prediction of the persistently high SHR trajectory

**Figure 4:**
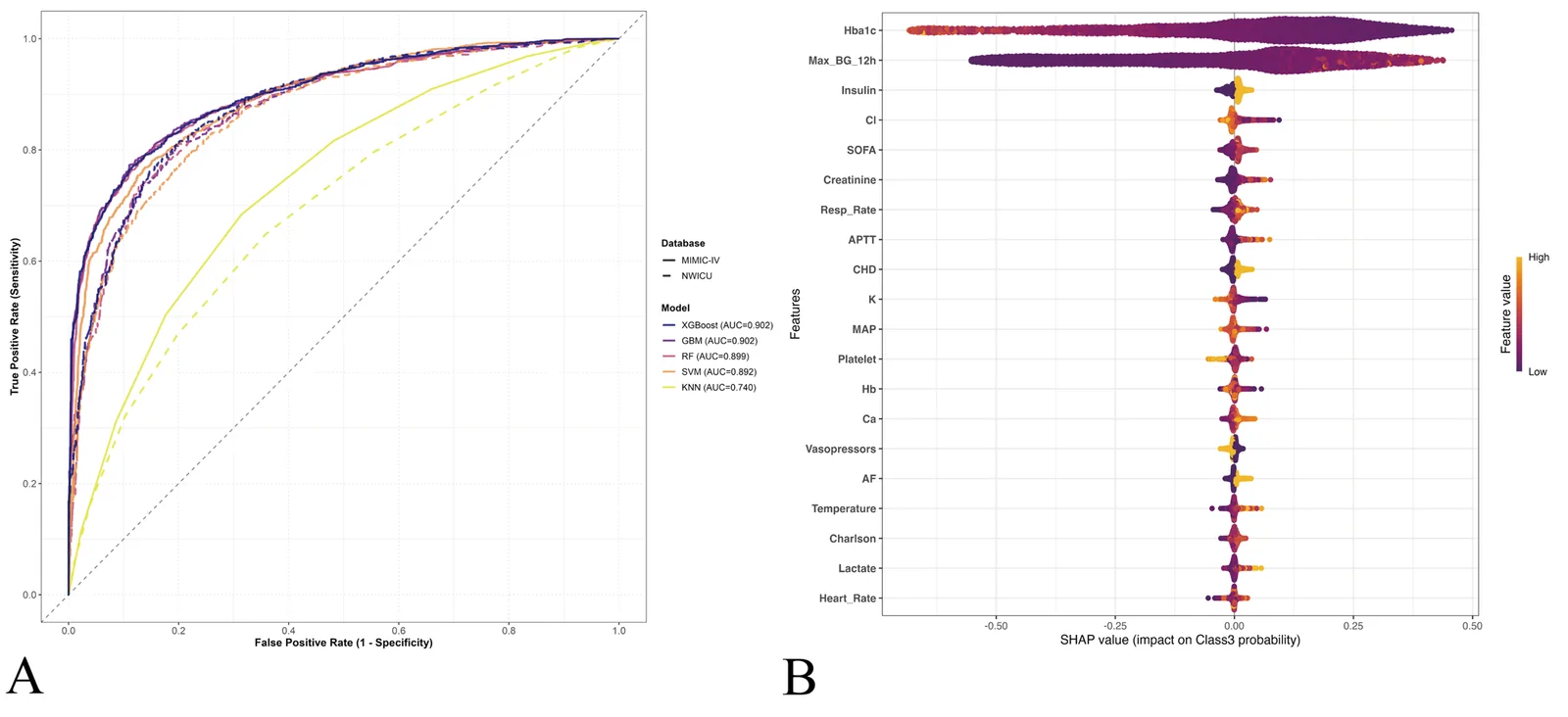
Performance and interpretation of machine-learning models for predicting membership in the persistently high SHR trajectory (Class 3). **(A)** Receiver operating characteristic curves for five models in the MIMIC-IV internal validation set and NWICU external validation cohort. Solid and dashed lines represent MIMIC-IV and NWICU, respectively. **(B)** SHAP summary plot for the final XGBoost model, showing each feature’s contribution to the predicted probability of Class 3 membership. AF, atrial fibrillation; APTT, activated partial thromboplastin time; AUC, area under the receiver operating characteristic curve; Ca, calcium; CHD, coronary heart disease; Cl, chloride; GBM, gradient boosting machine; Hb, hemoglobin; HbA1c, glycated hemoglobin; K, potassium; KNN, K-nearest neighbors; MAP, mean arterial pressure; MIMIC-IV, Medical Information Mart for Intensive Care IV; NWICU, Northwestern ICU database; RF, random forest; ROC, receiver operating characteristic; SHAP, SHapley Additive exPlanations; SHR, stress hyperglycemia ratio; SOFA, Sequential Organ Failure Assessment; SVM, support vector machine; XGBoost, extreme gradient boosting.

To identify patients likely to follow the persistently high SHR trajectory using early clinical information, machine-learning models were developed to predict Class 3 membership. Considering both linear and nonlinear feature-selection strategies, the final predictor set was derived from the intersection of LASSO- and Boruta-selected variables, covering glucose indices, comorbidities, severity scores, vital signs, laboratory measures, and early organ-support interventions (**Supplementary Figure 2**). Hyperparameters were optimized using repeated 10-fold cross-validation with three repeats within the MIMIC-IV training set, and the selected optimal settings are summarized in **Supplementary Table 4**. Among the five evaluated algorithms, XGBoost showed the best overall balance of discrimination and external performance and was selected as the final model. In the MIMIC-IV internal validation set, XGBoost achieved an AUC of 0.902, with performance comparable to GBM (AUC 0.902) and slightly higher than random forest (AUC 0.899), support vector machine (AUC 0.892), and K-nearest neighbors (AUC 0.740) (**Figure 4A**, **Supplementary Figure 2D**). In NWICU, XGBoost maintained good discrimination (AUC 0.882). SHAP analysis identified HbA1c and first-12-hour maximum glucose as the dominant predictors of Class 3 membership, consistent with the construction of SHR as a measure of acute glucose elevation relative to chronic glycemic background (**Figure 4B**). Beyond these SHR-defining variables, markers of illness severity, organ dysfunction, and early treatment intensity, including SOFA score, creatinine, respiratory rate, APTT, vasopressor use, and insulin therapy, also contributed to model predictions. An interactive web-based calculator implementing the final XGBoost classifier was developed and made publicly available at https://wangkangxing.shinyapps.io/shr-class3-calculator/.

## Discussion

In this dual-cohort study of critically ill patients with ASCVD, we identified three distinct SHR trajectories during the first 72 hours after ICU admission. The persistently high SHR trajectory (Class 3) was characterized by greater illness severity and the poorest 28-day survival in both the development and external validation cohorts. Its association with mortality remained evident after extensive adjustment and in the Day-3 landmark analyses restricted to patients surviving beyond 72 hours, including additional adjustment for glucose-monitoring frequency. These findings suggest that a persistently high SHR trajectory may identify a high-risk metabolic phenotype associated with greater clinical severity. Nevertheless, the trajectory should be interpreted as a prognostic marker rather than a direct measure or causal determinant of disease severity.

Previous studies have predominantly assessed SHR using a single admission measurement or a prespecified threshold. Recent cohort studies and meta-analyses have consistently associated elevated SHR with mortality and adverse cardiovascular outcomes in acute myocardial infarction, coronary artery disease, cardiac intensive care, ischemic stroke, and broader critically ill cardiovascular populations (9, 23–28). Our findings extend this evidence by demonstrating that the persistence and temporal pattern of relative hyperglycemia provide information beyond an isolated SHR value. The reproducibility of the three trajectory patterns in NWICU further supports their descriptive transportability, although differences between trajectory-based classification and single-value thresholds should be recognized.

SHR quantifies acute glucose elevation relative to the HbA1c-derived chronic glycemic background (3, 6). Accordingly, the high SHR observed in Class 3 reflects a comparatively large acute glucose deviation rather than simply a high absolute glucose concentration. Conversely, Class 2 had the highest prevalence of diabetes and the highest HbA1c level but followed a lower SHR trajectory, suggesting a substantial chronic hyperglycemic burden with a smaller relative acute deviation. Importantly, Class 2 should not be regarded as a “safe” phenotype. Although its fully adjusted association with 28-day mortality did not reach statistical significance, the point estimates generally indicated higher risk than Class 1, and the relatively small number of patients and outcome events in this class may have limited statistical power. Recent studies have also reported nonlinear or U-shaped associations between SHR and mortality among patients with diabetes, prediabetes, or acute heart failure (29–31). Therefore, SHR trajectories should be interpreted jointly with HbA1c, diabetes status, absolute glucose levels, and other indicators of chronic glycemic exposure.

Several mechanisms may explain the association between a persistently high SHR trajectory and adverse outcomes. Acute critical illness activates catecholamine, cortisol, inflammatory cytokine, and counter-regulatory hormone pathways, resulting in increased hepatic glucose production and insulin resistance (29, 32). Excessive relative hyperglycemia may aggravate oxidative stress, endothelial dysfunction, inflammatory activation, platelet aggregation, coagulation, and impaired microvascular perfusion. These effects may be particularly detrimental in patients with established ASCVD, whose vascular reserve is already compromised (29, 32). However, SHR may reflect both the biological consequences of metabolic dysregulation and the severity of the underlying illness. Moreover, observational risk associations do not imply that rapid normalization of glucose will improve outcomes. Recent randomized evidence and patient-level analyses have not demonstrated a consistent mortality benefit from universal intensive glucose control, while hypoglycemia remains an important concern (33–35). Current critical-care and diabetes guidelines therefore emphasize protocolized monitoring, avoidance of severe hyperglycemia and hypoglycemia, and individualized treatment rather than routine normalization to a narrow glucose range (36).

From a clinical perspective, serial SHR assessment could be incorporated into electronic ICU data systems using routinely available glucose and HbA1c measurements. A persistently high or rising SHR pattern could prompt earlier reassessment of illness severity, infection or ischemic burden, hemodynamic instability, nutritional exposure, medications, and the safety of glycemic management. Before the complete 72-hour trajectory becomes available, the machine-learning classifier developed in this study may provide an early estimate of the probability of Class 3 membership using information obtained within the first 24 hours. The web-based calculator facilitates research use of this model, but it is not intended to directly predict mortality or determine treatment. Glucose-monitoring intensity must also be considered because patients with more severe illness are tested more frequently and therefore have more opportunities for transient maxima to be recorded. Adjustment for the 72-hour glucose-measurement count did not materially alter the Class 3 association, but residual measurement-intensity bias cannot be excluded. Prospective studies using standardized sampling schedules or continuous glucose monitoring are needed to confirm the trajectory patterns and determine whether they improve clinical decision-making (36–38).

The subgroup analysis showed a statistically significant interaction between SHR class and heart failure on the multiplicative scale. This finding is biologically plausible because neurohormonal activation, impaired tissue perfusion, systemic inflammation, insulin resistance, and altered glucose utilization in heart failure may modify the relationship between acute glycemic stress and mortality. Recent studies have likewise associated elevated SHR with mortality and rehospitalization in heart failure populations (38, 39). Nevertheless, the interaction was derived from an observational subgroup analysis involving multiple comparisons and should be considered hypothesis-generating rather than evidence of a causal difference in treatment response. The machine-learning results were broadly consistent with the clinical analyses: HbA1c and first-12-hour maximum glucose were the dominant predictors of Class 3 membership, while severity, organ dysfunction, and early treatment variables provided additional predictive information. Although the final XGBoost model retained good discrimination in NWICU, its clinical utility, calibration across settings, and effect on patient outcomes require prospective evaluation.

This study has several strengths, including its large dual-cohort design, dynamic assessment of SHR across six clinically interpretable windows, external trajectory assignment, extensive covariate adjustment, multiple imputation, Day-3 landmark analyses, explicit adjustment for glucose-monitoring frequency, and development of an externally evaluated prediction model and research calculator. Several limitations should also be acknowledged. First, the retrospective observational design precludes causal inference and cannot eliminate residual confounding. Second, requiring HbA1c measurements may have introduced selection bias because HbA1c was more likely to be obtained in patients with suspected glycemic abnormalities. Third, using the maximum glucose value within each window remains susceptible to monitoring-intensity bias despite adjustment for measurement count. Fourth, trajectory assignment depends on the selected time windows, number of measurements, and modeling assumptions, while the relatively small Class 2 group reduced precision. Fifth, the heart-failure interaction was exploratory and was not evaluated on the additive scale. Finally, both databases were derived from US healthcare systems, and the machine-learning model and web calculator have not undergone prospective or interventional validation. These considerations support cautious interpretation and validation in more diverse, prospectively monitored populations.

## Conclusions

In critically ill patients with ASCVD, early SHR trajectories identified a high-risk relative glycemic stress phenotype in both MIMIC-IV and NWICU. This phenotype was characterized by acute glycemic elevation relative to HbA1c-defined background rather than chronic hyperglycemic burden alone, and was independently associated with increased 28-day, ICU, and in-hospital mortality. Dynamic SHR profiling may provide a useful framework for risk stratification and metabolic surveillance in cardiovascular critical illness. High SHR should be interpreted as a risk signal rather than as direct evidence supporting indiscriminate intensive glucose lowering. Prospective studies are needed to determine whether trajectory-informed metabolic monitoring can improve clinical decision-making and patient outcomes.

## Data Availability

The original contributions presented in the study are included in the article/**Supplementary Material**. Further inquiries can be directed to the corresponding author.

## Supplementary material

Supplementary Figure 1 Day-3 landmark survival analysis in the development and external validation cohorts. **(A)** Day-3 landmark Kaplan-Meier curves in the MIMIC-IV development cohort. **(B)** Corresponding curves in the NWICU external validation cohort. The post-landmark analysis included patients alive at 72 hours, with survival reset at day 3. Separate log-rank tests were performed for the 0–3-day and post-landmark periods. ICU, intensive care unit; MIMIC-IV, Medical Information Mart for Intensive Care IV; NWICU, Northwestern ICU database; SHR, stress hyperglycemia ratio.

Supplementary Figure 2 Feature selection, hyperparameter optimization, and performance evaluation of the machine-learning models. **(A)** Boruta feature-selection results. **(B)** LASSO cross-validation curve with the minimum-error and one-standard-error penalties. **(C)** Features selected by Boruta and LASSO. **(D)** Performance of the five models in the MIMIC-IV internal validation set and NWICU external validation cohort. AUC, area under the receiver operating characteristic curve; CV, cross-validation; GBM, gradient boosting machine; KNN, K-nearest neighbors; LASSO, least absolute shrinkage and selection operator; MCC, Matthews correlation coefficient; MIMIC-IV, Medical Information Mart for Intensive Care IV; NWICU, Northwestern ICU database; RF, random forest; SVM, support vector machine; XGBoost, extreme gradient boosting.

Supplementary Table 1 Model-fit and classification indices for candidate SHR trajectory models in the MIMIC-IV development cohort.

Supplementary Table 2 Comparison of eligible ASCVD ICU patients with and without available HbA1c measurements in MIMIC-IV.

Supplementary Table 3 Associations between SHR trajectory classes and ICU and in-hospital mortality in the development and external validation cohorts.

Supplementary Table 4 Hyperparameter tuning settings for the machine-learning models used to predict membership in the persistently high SHR trajectory (Class 3).
